# Marine natural product lepadin A as a novel inducer of immunogenic cell death via CD91-dependent pathway

**DOI:** 10.1007/s13659-023-00401-3

**Published:** 2023-10-02

**Authors:** Dalila Carbone, Carmela Gallo, Genoveffa Nuzzo, Giusi Barra, Mario Dell’Isola, Mario Affuso, Olimpia Follero, Federica Albiani, Clementina Sansone, Emiliano Manzo, Giuliana d’Ippolito, Angelo Fontana

**Affiliations:** 1 Institute of Biomolecular Chemistry, Consiglio Nazionale Delle Ricerche, Via Campi Flegrei 34, Pozzuoli, 80078 Naples, Italy; 2 Department of Biology, University of Naples “Federico II”, Via Cupa Nuova Cinthia 21, 80126 Naples, Italy; 3 Stazione Zoologica Anton Dohrn, Istituto Nazionale di Biologia, Ecologia e Biotecnologie Marine, University of Naples “Federico II”, Villa Comunale, 80121 Naples, Italy

**Keywords:** Immunogenic cell death, Natural products, Anticancer, Immunotherapy, Drug discovery

## Abstract

Immunogenic Cell Death (ICD) represents a mechanism of enhancing T cell-driven response against tumor cells. The process is enabled by release of damage-associated molecular patterns (DAMPs) and cytokines by dying cells. Based on molecular studies and clinical marker assessment, ICD can be a new target for cancer chemotherapy hitherto restricted to a few conventional anticancer drugs. In view of the development of small molecules in targeted cancer therapy, we reported the preliminary evidence on the role of the natural product lepadin A (**1**) as a novel ICD inducer. Here we describe the ICD mechanism of lepadin A (**1**) by proving the translocation of the protein calreticulin (CRT) to the plasma membrane of human A2058 melanoma cells. CRT exposure is an ICD marker in clinical studies and was associated with the activation of the intrinsic apoptotic pathway in A2058 cells with lepadin A (**1**). After the treatment, the tumour cells acquired the ability to activate dendritic cells (DCs) with cytokine release and costimulatory molecule expression that is consistent with a phenotypic profile committed to priming T lymphocytes via a CD91-dependent mechanism. The effect of lepadin A (**1**) was dose-dependent and comparable to the response of the chemotherapy drug doxorubicin (**2**), a well-established ICD inducer.

**Supplementary Information:**

The online version contains supplementary material available at 10.1007/s13659-023-00401-3.

## Introduction

Immunogenicity of neoplastic cells is a recognized target in the development of innovative cancer therapy. The use of checkpoint inhibitors or gene-engineered car-T cells have routinely entered in clinical operations [1, 2], whereas other approaches, such dendritic cell-based vaccines, need more time to be translated from the laboratory to the bedside [3]. Among the new research lines to elicit anticancer immune response, Immunogenic Cell Death (ICD) has recently raised interest as a potential mechanism to enhance the effects of conventional chemotherapy [4]. During ICD, cancer cells initiate specific pathways leading to the change of cell surface composition and release of damage-associated molecular patterns (DAMPs). These signals trigger recruitment of antigen-presenting cells (APCS) and result in activation of cytotoxic T lymphocytes-mediated immune response and ultimately death of cancer cells [5–7]. Calreticulin (CRT) and heat shock proteins (HSPs) expression on the surface of dying cells is considered one of the most common signals associated to ICD [8, 9]. CRT is the most significant calcium-binding chaperone in the endoplasmic reticulum (ER) lumen. Its amount significantly increases at the surface of cells triggered to apoptosis, thus modulating the process of their elimination. In response to the treatment with ICD inducers, the protein translocates on the outer surface of the cell membrane and acts as a de novo uptake signal for phagocytosis of CRT receptor (CD91)–positive cells, such as dendritic cells (DCs) and macrophages [10–12].

Although ICD has been studied with increased interest and the identification of single-ICD inducers is a progressively expanding field of investigation [12], to date only a limited number of conventional drugs, such as some anthracycline antibiotics and kinase inhibitors, are recognized ICD agents [13, 14]. Lepadins are cis‐fused decahydroquinoline (DHQ) marine alkaloids described for different biological activities, including cytotoxicity, tyrosine kinase inhibition and antiparasitic activity [15]. Starting from the development of an innovative screening platform for the identification of immunomodulatory compounds [16], we have recently reported that lepadin A (**1**) shows a cytotoxic effect in a panel of cancer cell lines along with the ability to induce maturation of mouse dendritic cells (DCs). The combination of this activity led us to put forward a potential role as ICD inducer for this natural product [17].

This work aims to investigate lepadin A (**1**) mechanism of action by evaluating the exposure of CRT as ICD marker in human melanoma A2058 cells and the consequent response of monocyte-derived dendritic cells (MoDCs) to the cancer cells treated with the marine alkaloid. For an assessment of the clinical potential to reverse a tumor-induced immunosuppressive microenvironment, we compared the activity of lepadin A (**1**) to the chemotherapy drugs doxorubicin (**2**), a known ICD inducer [18], and cisplatin (**3**), which is incapable of triggering translocation of CRT to the surface of dying cells [19, 20].

## Results

### Purification and cytotoxic activity of lepadin A (**1**)

**Fig. 1:**
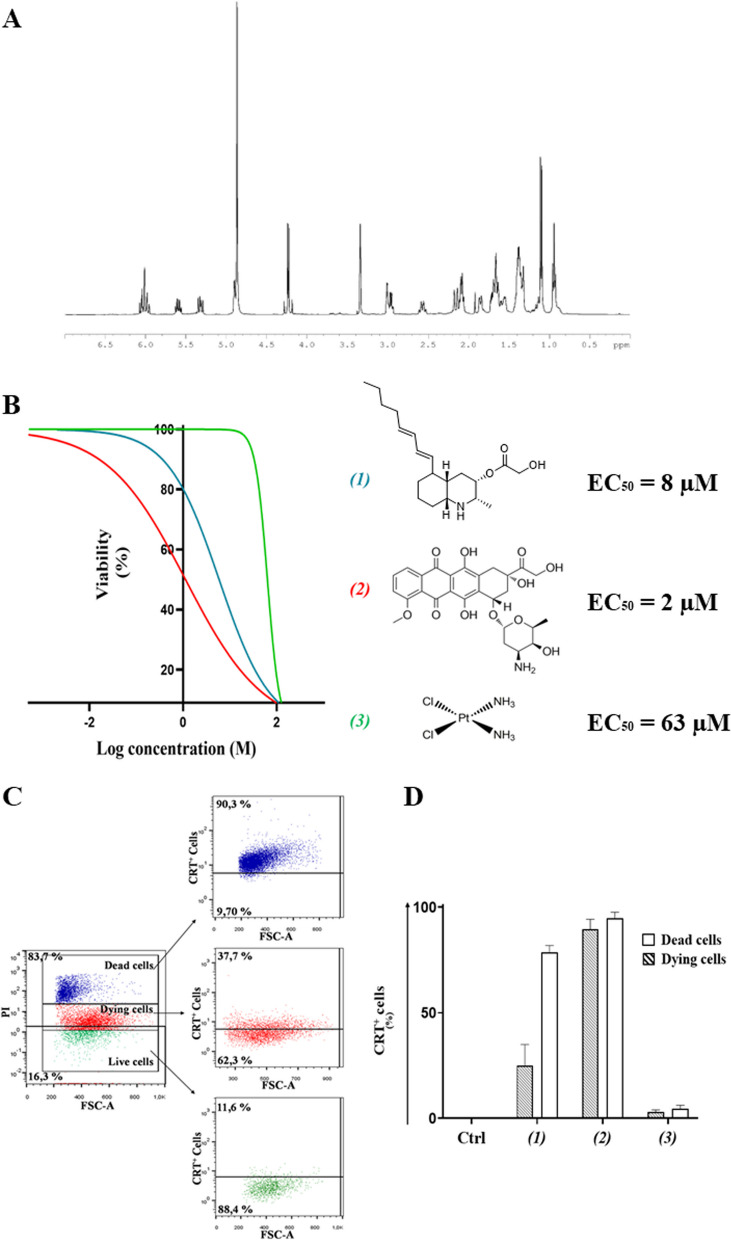
**A** 1H-NMR spectrum of lepadin A (**1**). The spectrum was recorded in CDCl_3_ at 600 MHz. **B** Cytotoxic activity of lepadin A (**1**), doxorubicin (**2**) and cisplatin (**3**) in the concentration range from 2 nM to 100 µM on human A2058 melanoma cells. A nonlinear regression analysis was performed for the estimation of the EC_50_ (50% of the effective concentration) reported alongside the chemical structures of the tested molecules. **C** Representative flow cytometry analyses with 8 μM lepadin A (**1**). Cells were gated according to forward (FSC) and viability (PI) signals. Selected populations were further selected on the fluorescence intensity due to the exposed CRT on the cell surface. Percentage refers to the fraction of CRT^+^ cells in each population. Blue = dead cells; Red = dying cells; Green = viable cells; **D** CRT exposure in A2058 cells treated with lepadin A (**1**), doxorubicin (**2**) and cisplatin (**3**). Percentage (*n* = 3) of CRT exposed on the surface of dead and dying cells after 24 h from the addition of compounds **1–3** at the EC_50_ concentrations. Ctrl = untreated cells. Statistical significance (****p < 0.0001) was established by One Way Anova

Lepadin A (**1**) was enriched from the extract of the ascidian *Clavelina lepadiformis* by Sephadex LH-20 in methanol and silica column with CHCl_3_/MeOH 9:1 (v/v) as previously described [17]. Final purification was performed on SPE-NH_2_ column by a gradient of isopropanol in *n*-hexane. The product was identified on the basis of data from NMR (Fig. 1A) in CD_3_OD and HR-ESI–MS (Additional file 2: Fig. S1).

### Cytotoxic activity of lepadin A (**1**) in human melanoma A2058 cells

A2058 cells were selected on the basis of the cytotoxicity of lepadin A (**1**) on a panel of 9 human cancer cell lines including lung cancer (LC), melanoma (Mel) and multiple myeloma (MM) [17]. The marine alkaloid (**1**) showed a dose-dependent activity with a response comparable to the anticancer drug doxorubicin (**2**). The two compounds shared similar EC_50_, namely 8 µM for **1** and 2 µM for **2**, but the threshold dose levels were significantly different (0.2 µM for **1** and 0.05 µM for **2**). In the same concentration range, the profile activity of cisplatin (**3**) indicated a sharp increase of the slope curve that matched a significantly higher EC_50_ (63 µM) and a threshold limit hundred times greater than lepadin A (**1**) and doxorubicin (**2**), which means it takes a larger dose to begin exerting effects.

### Induction of CRT exposure in A2058 cells

CRT is exposed at the early stage of ICD and dictates the immunogenicity of cancer cells [9]. By immunocytochemistry methods and established protocol of flow cytometry [10, 21, 22], we measured CRT exposure in A2058 cells after treatment with compounds **1–3** at EC_50_ concentrations (Fig. 1B). Following the gating strategy described in the Supporting Information (Additional file 2: Figs. S2 and S3), cells were labeled with an anti-CRT monoclonal antibody followed by staining with FITC-conjugated secondary antibody and the vital dye propidium iodide (PI). Fluorescence intensity due to PI progressively increased from viable to dying cells, reaching the highest value in dead cells. This difference in fluorescence intensity was used to identify alive (no fluorescence), dying (low fluorescence) and dead cells (high fluorescence) and allowed to establish the exposure of CRT in each population (Fig. 1C). As shown in Fig. 1D, FITC-marked CRT was not detected in the viable cells (Ctrl), whereas the fraction of CRT + was very high with doxorubicin (**2**) (above 90%) and lepadin A (**1**) (70%) in the population of dead cells. Significant presence of CRT in the membrane of dying A2058 cells also occurred with doxorubicin (**2**) (about 90% of the whole population) and lepadin A (**1**) (about 40% of the whole population), which is in agreement with the translocation of the protein in an early phase of the anti-cancer mechanism. On the other hand, no signal for CRT was detected after treatment with 63 µM cisplatin (**3**) despite the intrinsic toxicity of this drug.

### Flow Cytometry and confocal analysis of CRT translocation induced by lepadin A (**1**) in A2058 cells

**Fig. 2:**
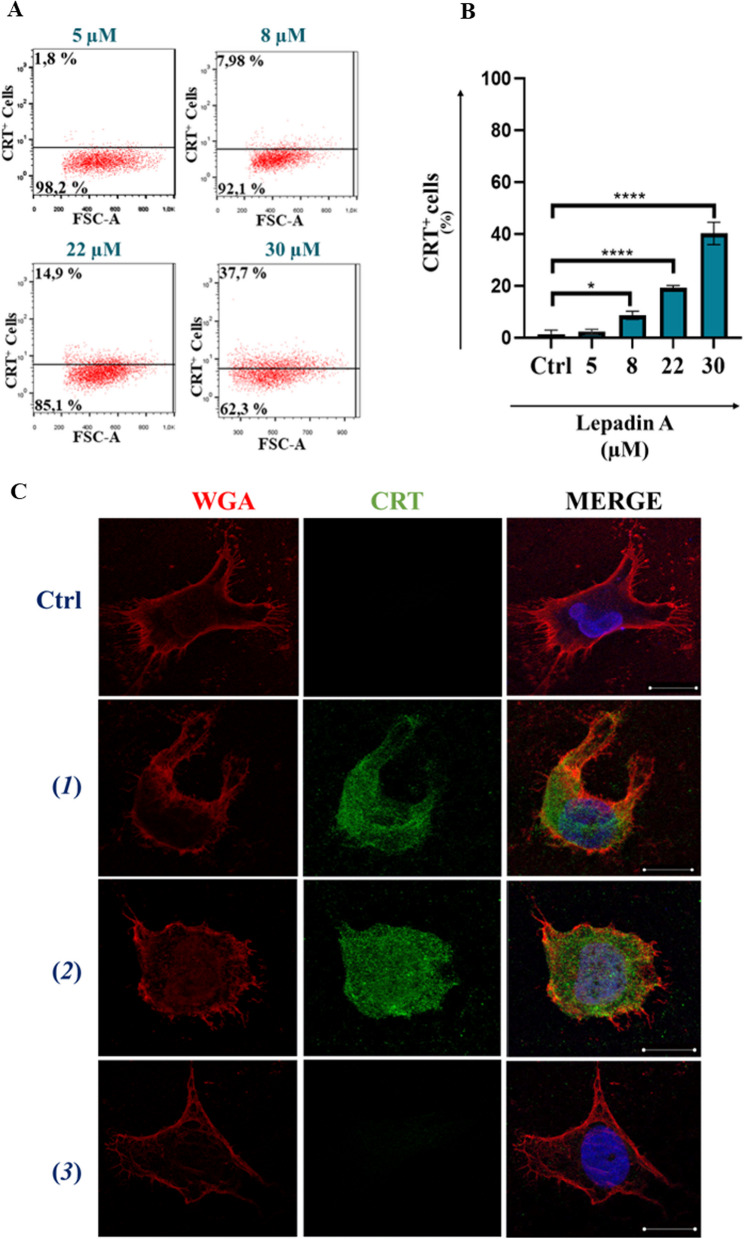
Effect of lepadin A (**1**) on A2058 melanoma cells. **A** Representative experiment of scatter (FSC-A) versus CRT fluorescence dot plots related to dying cells after treatment with increasing concentration (from 5 to 30 µM) of lepadin A (**1**). **B** Percentage (*n* = 3) of CRT exposed on the surface of dying cells after 24 h by different concentrations of lepadin A (**1**). Ctrl = untreated cells. Statistical significance (*p < 0.5; ****p < 0.0001) was established by One Way Anova. **C** Representative confocal microscopy images of A2058 cells treated with lepadin A (**1**), doxorubicin (**2**) and cisplatin (**2**). Ctrl = untreated cells. Cells were stained with DAPI (blue) for nuclei, Calreticulin monoclonal antibody conjugated at Alexa fluor 488 secondary antibody (green) for CRT, and wheat germ agglutinin (WGA) (red) for plasma membranes. Merged confocal images or individual channels are shown. Images were acquired on a Zeiss LSM 700 confocal microscope, with a 63X objective (NA 1.4) and a zoom of 1.5 and 2 respectively. Scale bar 5 μm

To corroborate the ICD mechanism, A2058 cells were treated with increasing concentrations of lepadin A (**1**) from 8 to 30 µM and the dying cells population was selected by flow cytometry with the same gating strategy discussed above (Fig. 2A). The analysis demonstrated a dose-dependent increase of cells exposing CRT on the outer membrane, with an effect linearly related to the concentration of the marine alkaloid (**1**) (Fig. 2B). Membrane translocation of CRT on the plasma membrane was visualized by confocal laser scanning microscopy (CLMS) and labelling of the cells with anti-CRT antibody and biotinylated anti-wheat germ agglutinin (WGA) antibody for membrane lectins [23]. After staining with diamidino-2-phenylindole (DAPI) for nucleic acids, localization of FITC-marked CRT in the cell membranes labeled in red with streptavidin demonstrated the homogeneous distribution of the protein on the cell surface after treatment with lepadin A (**1**) and doxorubicin (**2**). On the other hand, in agreement with the flow cytometry results, the CRT signal was not detectable in the controls and with cisplatin (**2**) (Fig. 2C). Confocal images also pointed out differences in the cell morphology due to the treatments. In controls (untreated samples) and with cisplatin (**2**), A2058 cells showed branched and irregularly squared shapes with centrally placed oval nucleus. Doxorubicin (**2**) induced change to a rounded morphology that agrees with previous observations [18], whereas the effect of lepadin A (**1**) was correlated to the presence of oval or bucket-shaped cells featured by variably processes of shrinkage and blebbing.

### Differential gene expression induced by lepadin A (**1**) in A2058 cells

**Fig. 3:**
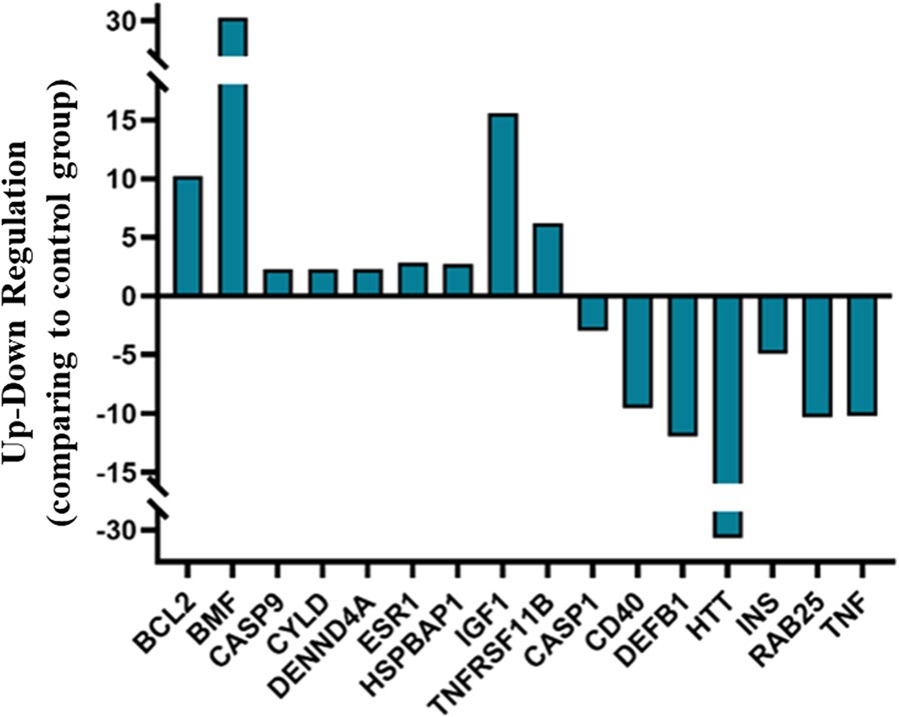
Differential expression analysis of genes related to cellular death mechanisms after treatment of A2058 cells with lepadin A (**1**). Bar plot comparing gene expression fold changes identified by microarray analysis. The x axis denotes the differentially expressed genes and the y axis represents the fold difference for mRNA levels in treated and untreated samples

To gain further information on the activity of the marine alkaloid, we performed a Profiler PCR Array Gene Expression Analysis for 84 key genes related to central mechanisms of cellular death such as apoptosis, autophagy, and necrosis. Compared with those in the untreated group, lepadin A (**1**) induced the differential expression of 16 genes, of which 9 were upregulated and 7 downregulated (|log2FC|> 1, p < 0.05) (Additional file 1). Notably, almost all differentially expressed genes were related to PCD progression dependent on ICD mechanism (Fig. 3). The most upregulated genes were BCL2 and BCL2 modifying factor (BMF), which are critical factors for intrinsic mitochondrial apoptosis signalling pathway [24, 25]. The differential analysis also showed upregulation of caspase-9 expression that is crucial in the downstream activation of caspase-3 in ICD signalling [26], while caspase-1 that is involved in ICD-independent PCD was downregulated [27]. Overexpression of the tumor suppressor deubiquitinase CYLD, DENND4A and Estrogen receptor α (ESR1) were also coherent with the activation of PCD [28–30]. Moreover, the increased levels of TNFRSF11B, ESR1, IGF1 and downregulation of RAB25, a member of the RAS oncogene family, resulted in line with the few reports on genes involved in the immunogenic modulation pathway [31].

### Activation of human Dendritic cells in co-cultures with A2058 cells treated with lepadin A (**1**)

**Fig. 4:**
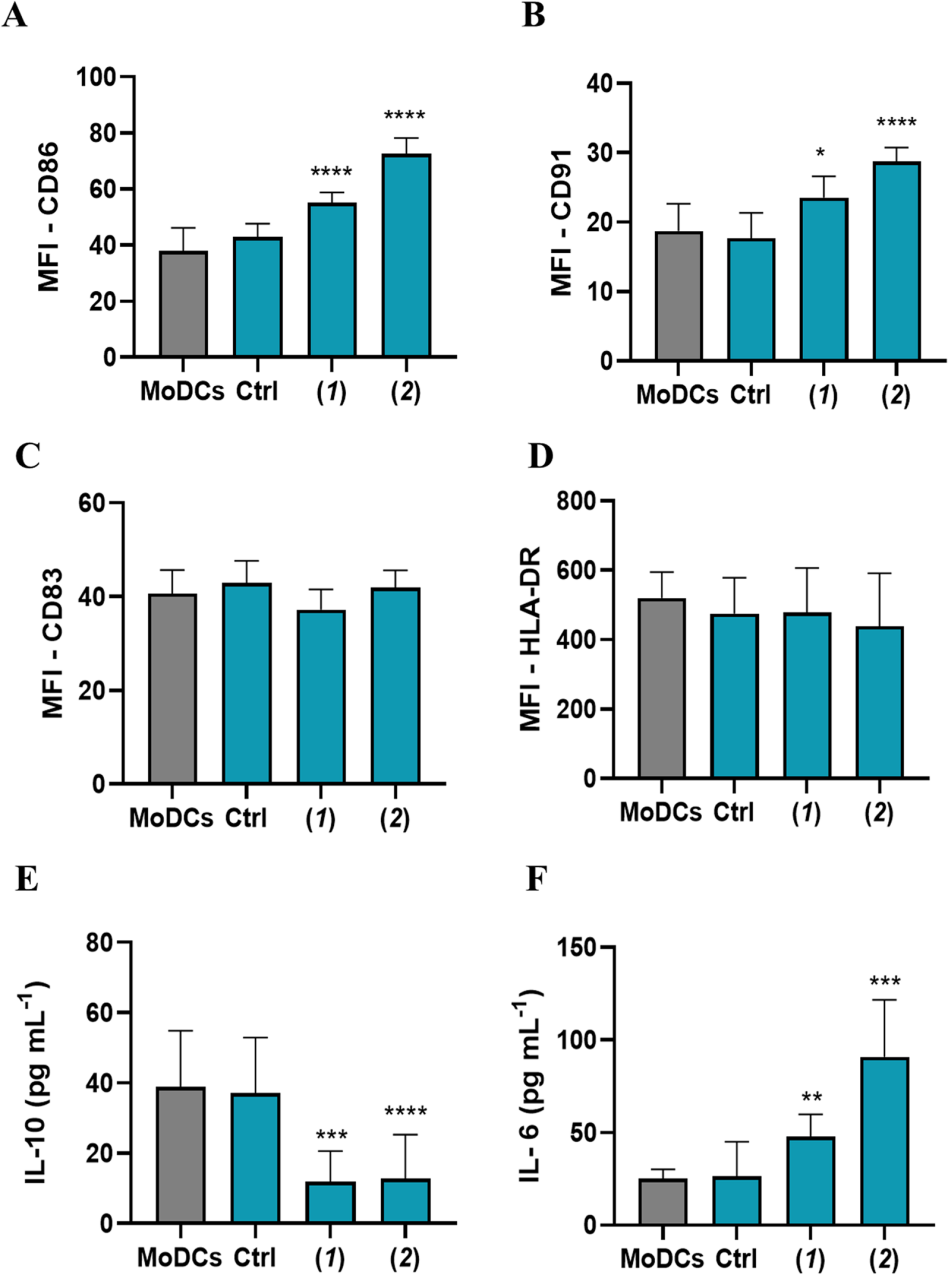
Phenotypic analysis of MoDCs in co-culture with A2058 cells (*n* = 6). **A**–**D** Flow cytometry analysis represent the Mean Fluorescence Intensity (MFI) of surface expression of CD86, CD91, CD83 and HLA-DR; **E**, **F** IL-10 and IL-6 cytokine levels (pg/mL^−1^) measured by ELISA assay in the supernatant. MoDCs = dendritic cells. Ctrl = MoDCs cocultured with untreated A2058 cells. (**1**) = MoDCs cocultured with A2058 cells treated with lepadin A (**1**). (**2**) = MoDCs cocultured with A2058 cells treated with doxorubicin (**2**). Statistical significance (*p < 0.5; ***p < 0.001; ****p < 0.0001) was established by One Way Anova

The involvement of DCs in the immune response triggered by ICD is reported in different studies that point out the crucial role of this mechanism in the tumour microenvironment (TME) [32]. To verify this, we tested the response of MoDCs from healthy human donors in co-cultures with A2058 cells treated with lepadin A (**1**). Cells untreated or treated with doxorubicin (**2**) were used as negative and positive controls, respectively. As shown in Fig. 4A–D, both lepadin A (**1**) and doxorubicin (**2**) did not change surface MHC II but increased the surface occurrence of the costimulatory molecule CD86 and lipoprotein receptor related protein 1 (LRP1, also known as CD91) in comparison with MoDCs co-cultured with untreated A2058 cells. Low CD86 has been reported in inadequately matured DCs that induce T-cell tolerance [33], while recognition of CRT on cancer cells by CD91 on macrophages and DC promotes phagocytosis of apoptotic cells [34] and priming of T-helper (Th) lymphocytes [35]. In line with these findings, we found no variation for CD83 that is highly expressed on mature DCs but is implicated in immune suppressive responses [36]. In further support to the upregulation of key effectors for tumour surveillance, lepadin A (**1**) increased synthesis of IL-6 and reduced the secretion of the immunosuppressive cytokine IL-10 in the supernatants of the co-cultures with DCs, while did not affect the levels of IL-1β and TNFα (Additional file 2: Fig. S4). Notably, this cytokine profile was identical to that observed with doxorubicin (**2**) while there was no change in cytokines without treatment of the cancer cells.

**Fig. 5:**
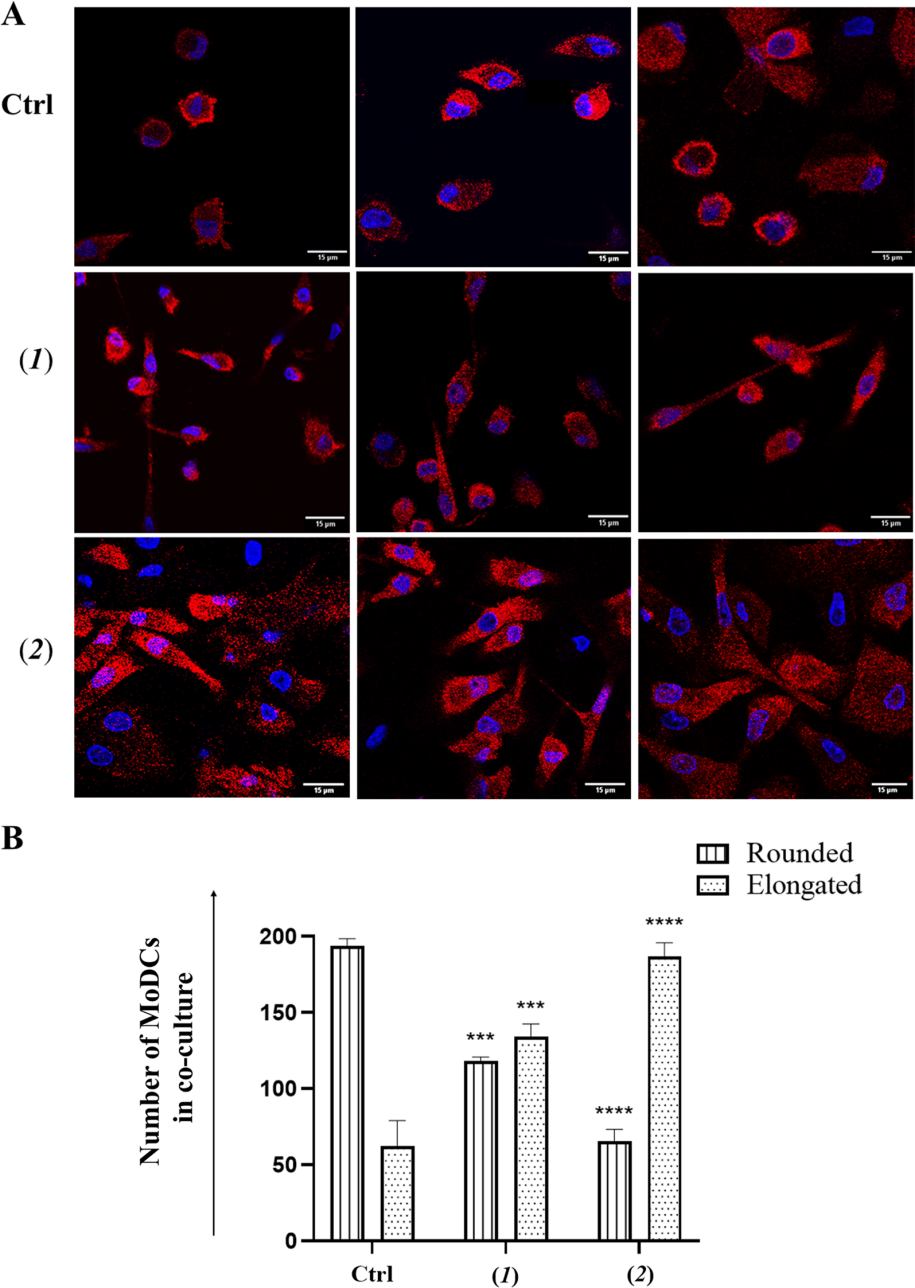
Morphological variation of MoDCs in co-culture with A2058 cells. **A** Representative confocal microscopy images of MoDCs from cocultures with A2058 cells treated with lepadin A (**1**), doxorubicin (**2**). MoDCs were selectively stained in red with HLA-DR monoclonal antibody conjugated with Alexa Fluor 647 secondary antibody. DAPI (blue) was used for nuclei. Merged confocal images or individual channels are shown. Images were acquired on a Zeiss LSM 700 confocal microscope, with a 63X objective (NA 1.4) and a zoom of 1.5 and 2 respectively. Scale bar 15 μm. **B** Cell number of MoDCs with rounded and elongated shape. Cell counting was performed on different fields (*n* = 2) of five different samples*.* Labels on the horizontal axis (category) are the same as Fig. 4. Statistical significance (***p < 0.001; ****p < 0.0001) was established by One Way Anova

Phenotypic activation linked to morphological variation of MoDCs was confirmed by immunofluorescence analysis. Staining of MoDCs with MHC class II antibody (HLA—DR) showed cells with different morphology in co-cultures with untreated A2058 cells or with A2058 cells treated with doxorubicin (**2**) and lepadin A (**1**). In the first case, cells have a prevalently rounded shape. In contact with A2058 cells treated with doxorubicin (**2**), MoDCs acquired a more elongated shape, which is an index of cell maturation. In the presence of cells treated with lepadin A (**1**), MoDCs showed both rounded and elongated shapes (Fig. 5A, B).

## Discussion and conclusion

The extension of the concept of immune surveillance to immune editing has introduced a more accurate view of the many facets of immune system–tumor interactions. Immunogenic cell death (ICD) involves a regulated mechanism of T cell response that is driven by both specific tumor-associated antigens and delivery of immunostimulatory molecules, such as membrane markers and soluble mediators, from the dying cells in a defined temporal sequence. Such signals are not normally expressed by cancer cells but can be induced by a few cytotoxic drugs.

Here we have reported that the marine alkaloid lepadin A (**1**) is an ICD inducer. The natural product triggers dose-dependent translocation of CRT from the lumen of ER to the membrane of the cancer cells, which is one of the most specific mechanisms involved in ICD [37]. Cancer cells lacking CRT expression are not efficiently engulfed by APCs, suggesting that CRT exposure is crucial for phagocytosis of the dying cells [38]. In experimental mouse vaccination protocols, CRT knockdown reduces the cancer cells immunogenicity and inhibits the ability to induce a protective immune response that can restored by administration of recombinant CRT [39]. This mechanism depends on the activity of CRT to facilitate tumor antigen transfer to DCs and antigen cross-presentation to T cells [40]. We showed that, after treatment with lepadin A (**1**), A2058 melanoma cells induced maturation of DCs with release of IL-6, reduction of the immunosuppressive IL-10 and upregulation of the costimulatory molecules CD86 and CD91, allowing for a phenotypic profile committed to promote T-cells response. CD91 is a recognized receptor of CRT and other immunogenic DAMPs on APCs. Specifically, engagement of CD91 by CRT triggers an intracellular signalling pathway to activate NF-Kβ in APCs and ultimately prime Th17 response with IL-6 having a pivotal role by inhibiting the generation of Treg cells [35]. Binder and coworkers reported that mice lacking this protein on DCs show higher tumour incidence due to inability to mount effective immune surveillance [41]. The same authors showed that the polymorphism of CD91 gene affects the binding of the protein with the ligands and correlates with the immune responses of patients affected by lung squamous cell carcinoma and cutaneous melanoma [41].

**Fig. 6:**
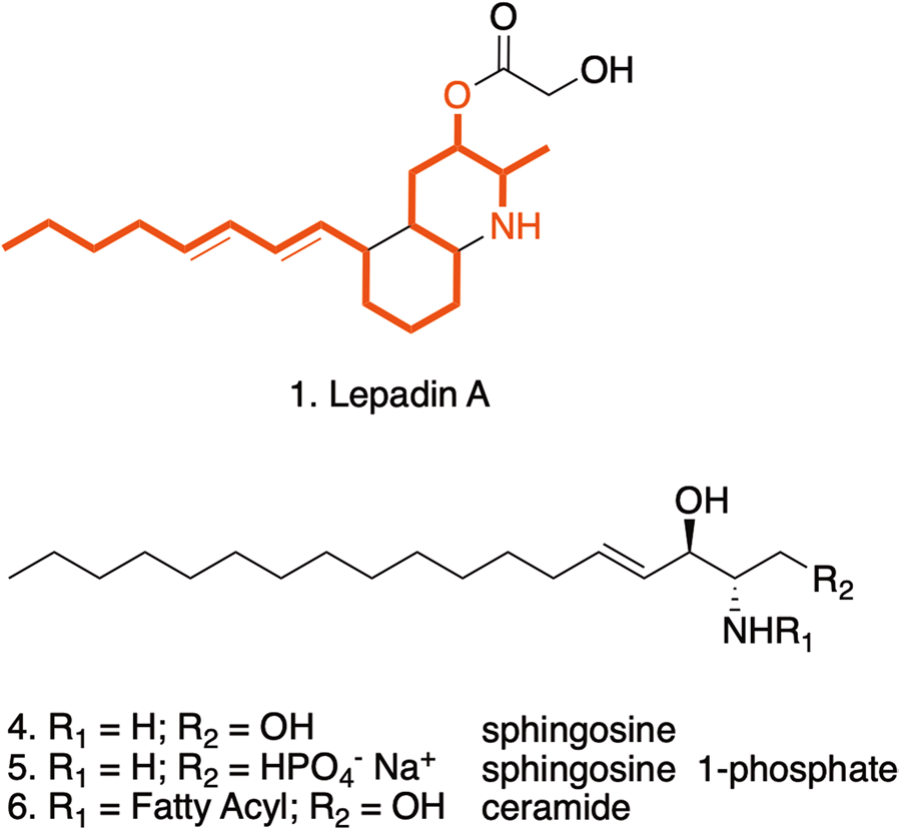
Structural analogies of lepadin A (**1**) with sphingosine (**4**) and its derivatives sphingosine 1-phosphate (***5***) and ceramides (***6***) reported in the intracellular signaling associated to ICD. The red notation highlights the C18-backbone and the amino-alcohol function of sphingosine that is preserved in lepadin A

The activity of lepadin A (**1**) was comparable to that of doxorubicin (**2**) that has been target of several clinical studies as ICD-eliciting agent [42, 43]. Lepadin A (**1**) can be envisioned as a sphingoid base-like compound [44] with the amino-alcohol function embedded within a isoquinoline ring, and this might account for its biological activity (Fig. 6). Sphingolipids have been characterized as key intracellular mediators of ICD and have been linked to the mechanism of cell death induced by anthracyclines [45]. In particular, ceramide (Cer) and sphingosine-1-phosphate (S1P) could regulate the activity of the anthracycline doxorubicin in multiple cancer types [46, 47]. The structural analogies of lepadin A (**1**) backbone to these molecules could suggest a similar mechanism of action, involving the inhibition of the sphingosine kinases (SphKs) that enhances CRT translocation. Apoptotic cells increased in presence of CRT on the outer surface of plasma membranes. Differential expression of genes related to regulated cell death programs suggests that lepadin A (**1**) can activate the intrinsic apoptotic pathway and deregulation of genes linked to immunogenic cell stress [31]. This mechanism seems to differ from that of doxorubicin (**2**) that induces apoptosis of cancer cells by extrinsic pathway in a caspase-dependent manner [48].

In conclusion, the study indicates that the marine natural product lepadin A (**1**) causes apoptosis and death of human melanoma A2058 cells. The effect is associated to exposure of CRT on the membrane of the cancer cells, which triggers maturation and activation of DCs by a CD91-dependent pathway. In combination with the ability of lepadin A (**1**) to elicit direct DC maturation at sub-lethal doses [13], the present study highlights the potential of the marine alkaloid (**1**) as an immunogenic anticancer drug that combines the systemic immunomodulatory activity mediated by DC stimulation with the protective effect deriving by the ICD mechanism on cancer cells. This result needs in vivo studies in syngeneic mouse models but promises to overtake the paradigm of the maximum tolerated dose that drives anti-cancer research in absence of any consideration for potentiating immunomodulatory effects [49].

## Experimental section

### Chemical compounds

Cisplatin was purchased from Accord Healthcare (London, United Kingdom). Doxorubicin and sulforhodamine B (SRB) assay Kit (Abcam ab235935) were obtained from Abcam (Cambridge, United Kingdom). Lepadin A (**1**) was isolated with a purity higher than 99% from the extract of the ascidian *Clavelina lepadiformis* [17].

### Cancer cell lines

The human cell lines A2058, CALU-1, CALU-3, HCC827, MALME-3 M, A375 were purchased from the American Type Culture Collection (ATCC); 3 multiple myeloma lines, KMS-12, RPMI 8226, JJN-3 were purchased from the German Collection of Microorganisms and Cell Cultures (DSMZ). All cells were cultured as previously reported by [16].

### MoDCs culture

Monocyte-derived dendritic cells (MoDCs) were obtained by differentiation from human peripheral blood of healthy volunteers collected at Umberto I Hospital of Nocera Inferiore, Salerno (Italy) as described before [50].

### Determination of EC_50_ value

Cytotoxicity was tested by the SRB assay kit. Human melanoma A2058 cells were transferred to 96-well plates at a concentration of 1 × 10^4^ cells per well and treated with lepadin A (**1**), doxorubicin (**2**) and cisplatin (**2**) from 2 nM to 100 µM starting from DMSO stock solutions of each compound at concentration of 5 mg/mL. After 24 h, cells were fixed and stained according to manufacturer’s instructions. The optical density was determined at 565 nm and cytotoxicity was calculated as reported by [16]. Spectrophotometric measurements were performed using EZ Read 2000 microplate readers (Biochrom, Cambridge, United Kingdom).

### Flow cytometric analysis of CRT

A2058 cells (1.4 × 10^5^) were plated into 6-well plates and treated the day after with lepadin A (**1**), doxorubicin (**2**) and cisplatin (**2**) at EC_50_ concentrations for 24 h. Cells were washed by 200 µL of 1 M phosphate buffer saline (PBS) containing 1% BSA and 0.1% NaN_3_ and stained with 100 µL blocking buffer (PBS with 1% BSA, 0.1% NaN_3_ and 20% FBS) for 15 min at 4 °C to avoid non-specific signals. Samples were then incubated for 60 min at 4 °C in the dark with 3 µg/mL of calreticulin monoclonal primary antibody (1G6A7—Invitrogen, Thermo Fisher Scientific) in PBS, followed by washing and incubation for 30 min at 4 °C in the dark with 25 µL of PBS containing 5 µg/mL of FITC-Donkey anti-Mouse IgG (H + L) secondary antibody (A21202—Invitrogen, Thermo Fisher Scientific) and then with 1 µg/mL of Propidium Iodide Solution (Invitrogen, Thermo Fisher Scientific, Waltham, MA, USA) at room temperature for 5 min. Isotype-matched IgG antibodies were used as controls.

### Immunofluorescence

A2058 cells (1.4 × 10^5^) and MoDCs (5 × 10^5^) were transferred to glass 6-well plates covered by 12-mm coverslips (Knittel glass, Braunschweig, Germany) and incubated for 24 h. After fixation with 4% paraformaldehyde for 30 min, cells were washed by 500 µL PBS and treated with Blocking Solution (PBS with 0.5% BSA, 50 mM NH_4_Cl and 0.02% NaN_3_) without saponin for 30 min at room temperature to pick up the membrane signal and not permeabilizing the cells. A2058 cells were stained with 1 μg/mL of biotinylated wheat germ agglutinin (WGA) lectin and 10 μg/mL of calreticulin monoclonal primary antibody (1G6A7—Invitrogen, Thermo Fisher Scientific) for 1 h. MoDCs were incubated over night with 20 μg/mL of HLA-DR Monoclonal Antibody (SCO6-78, Invitrogen). Cells were then washed by 1 mL PBS and incubated with streptavidin DyLight™ 549 (1:5000), FITC-Donkey anti-Mouse IgG (H + L) (1:500; A-21202—Invitrogen, Thermo Fisher Scientific) and Alexa Fluor 647 Goat anti-Rabbit IgG (H + L) (1:400; A-21245—Invitrogen, Thermo Fisher Scientific) secondary antibodies for 30 min at room temperature. Finally, coverslips were stained for 5 min with 25 µL 4',6-diamidin-2-fenilindolo (DAPI) (1:1000; 94,403—Sigma-Aldrich, Saint Luis, USA), washed again and placed on a slide microscope with Mowiol® 4–88 reagent (Sigma-Aldrich, Saint Luis, USA). All samples were analysed under a confocal laser microscope (Zeiss LSM 700; Carl Zeiss, Gottingen, Germany) by using a 63 × oil-immersion objective (1.4 NA).

### Gene expression analysis

A2058 cells (1.4 × 10^5^) were incubated with 22,3 µM lepadin A (**1**) for 3 h and then lysed by addition of 1 mL TRizol™ reagent (Invitrogen, Thermo Fisher, Waltham, MA, USA). RNA was isolated following the manufacturer instructions and quantified by a NanoDrop 1000 Spectrophotometer (Thermo Fisher Scientific, Waltham, MA, USA). RT2 First Strand Kit (Qiagen Hilden, Germany) was used for the reverse transcription reaction and gene expression patterns were analysed by RT^2^ Profiler PCR Array "Human Cell Death Pathway Finder 384 HT" (*PAHS-212Z*, SA Biosciences) according to the recommended protocols. Data (Ct-values) were analysed as previously reported [51]. Values were considered significant if greater or lower than 2.0-expression ratios compared to controls.

### Co-culture of A2058 cells and dendritic cells

After harvesting, iMoDCs were immediately transferred to 24-well plates in RPMI medium and co-cultured for 24 h in a 20:1 ratio with A2058 cells (control) or A2058 cells previously treated with 22 µM lepadin A (**1**) or with 2 µM doxorubicin (**2**). Flow cytometry analysis was performed by staining with CD86 antibody (PE-Vio770, Clone REA968, Invitrogen, Thermo Fisher Scientific), CD83 (APC, Clone REA714, Invitrogen, Thermo Fisher Scientific), CD91 (PE, Clone A2MR-a2, Invitrogen, Thermo Fisher Scientific), HLA-DR (FITC, Clone L243, Biolegend) and Fixable Viability Stain 780 (APC-Vio770, BD Horizon). The viable HLA DR^+^ cell population was selected for surface expression markers analysis according to the gating strategy reported in the Supporting Information (Additional file 2: Fig. S5). All data were acquired by a MACSQuant® Analyzer 16 (Miltenyi Biotec, California, USA) and analysed by FlowJo 9 Software (Tree Star, Inc., Ashland, OR USA).

### ELISA

IL-6, IL-10, TNFα, IL-1β were measured in duplicate in the supernatants at 24 h by commercial kits (Thermo Fisher Scientific, Waltham, MA, USA) following the manufacturer instructions. The absorbance at 620 and 450 nm was quantified by EZ Read 2000 (Biochrom Ltd, Harvard bioscience) spectrophotometer and converted to pg/mL according to the standard curve generated with a five-parameter logistic curve fit.

### Statistical analysis

Statistical analysis was performed by GraphPad Prism 8.00 software (GraphPad Software, San Diego, CA, USA). Half maximal effective concentration (EC_50_) was calculated by Non-Linear regression analysis and EC_50_ shift function using the GraphPad Prism software.

### Supplementary Information

**Additional file 1.** Excel file reporting raw microarray data on gene expression analysis carried out on A2058 melanoma cells.

**Additional file 2: Figure S1**. MS spectrum of lepadin A (**1**). The analysis was carried in positive ion mode. m/z 335, 23 (M + Na +). **Figure S2.** Exposure of CRT on A2058 cell surface to the various treatments. CRT-specific fluorescence is plotted for each gated cells (dead = blue, dying = red, live = green); **A** A2058 cells treated with doxorubicin (**2**) at EC_50_ concentration (2 µM). **B** A2058 cells treated with Cisplatin (**3**) at EC_50_ concentration (63 µM). **C** A2058 cells treated with lepadin A (**1**) at EC_50_ concentration (8 µM). **Figure S3.** Gating strategy of CRT exposure. Cells were stained with 3 µg/mL of calreticulin primary antibody, with 5 µg/mL of secondary antibody (FITC) and then, with Propidium Iodide (PI). Plot A represent the physical population (SSC-A, FSC-A) corresponding to untreated A2058 cells. Plot B represents the exposure of CRT on gated A2058 of plot A, while plot C reports the PI staining. Live, dying and dead cells were gated in Plot C according to PI fluorescence intensity. CRT + cells (D-F) were then selected from each population of plot C. **Figure S4:** IL-1β and TNFα cytokine production (pg/mL^-1^) measured by ELISA assay. MoDCs = dendritic cells. Ctrl = MoDCs cocultured with untreated A2058 cells. (1) = MoDCs cocultured with 22 µM concentration of lepadin A (**1**) pre-treated A2058 cells; (2) = MoDCs cocultured with EC50 concentration of doxorubicin (**2**) pre-treated A2058 cells. Statistical significance (***p < 0.001, ****p < 0.0001) was established by One Way Anova. **Figure S5.** Gating strategy of MoDCs surface expression markers. Plot A represent the physical population (SSC-A, FSC-A) corresponding to MoDCs cocultured with A2058 cells. Plot B represents the exposure of HLA-DR on gated cells of plot A, MoDCs were HLA-DR + while A2058 cells were HLA-DR -. Plot C reports the Viability staining of MoDCs. CD91, CD86, CD83 (D-F) were then selected from plot C.

## Data Availability

All data generated or analyzed during this study are available in this published article and its Additional files.
