# Supplementary material for: Marine natural product lepadin A as a novel inducer of immunogenic cell death via CD91-dependent pathway
Source: Nat Prod Bioprospect. 2023 Oct 2;13(1):34. doi: 10.1007/s13659-023-00401-3 (PMC10542626; doi:10.1007/s13659-023-00401-3)

Supporting Information

**Marine Natural Product Lepadin A as a Novel Inducer of Immunogenic Cell Death via CD91-dependent pathway**

Dalila Carbone^[a]^, Carmela Gallo^*[a]^, Genoveffa Nuzzo^[a]^, Giusi Barra^[a]^, Mario Dell’Isola^[a]^, Mario Affuso^[b]^, Olimpia Follero^[a]^, Federica Albiani^[b]^, Clementina Sansone^[c]^, Emiliano Manzo^[a]^, Giuliana d’Ippolito^[a]^, Angelo Fontana ^[a] [b]^

**Figure S1:** MS spectrum of lepadin A (***1***). The analysis was carried in positive ion mode. m/z 335, 23 (M+Na+).

***
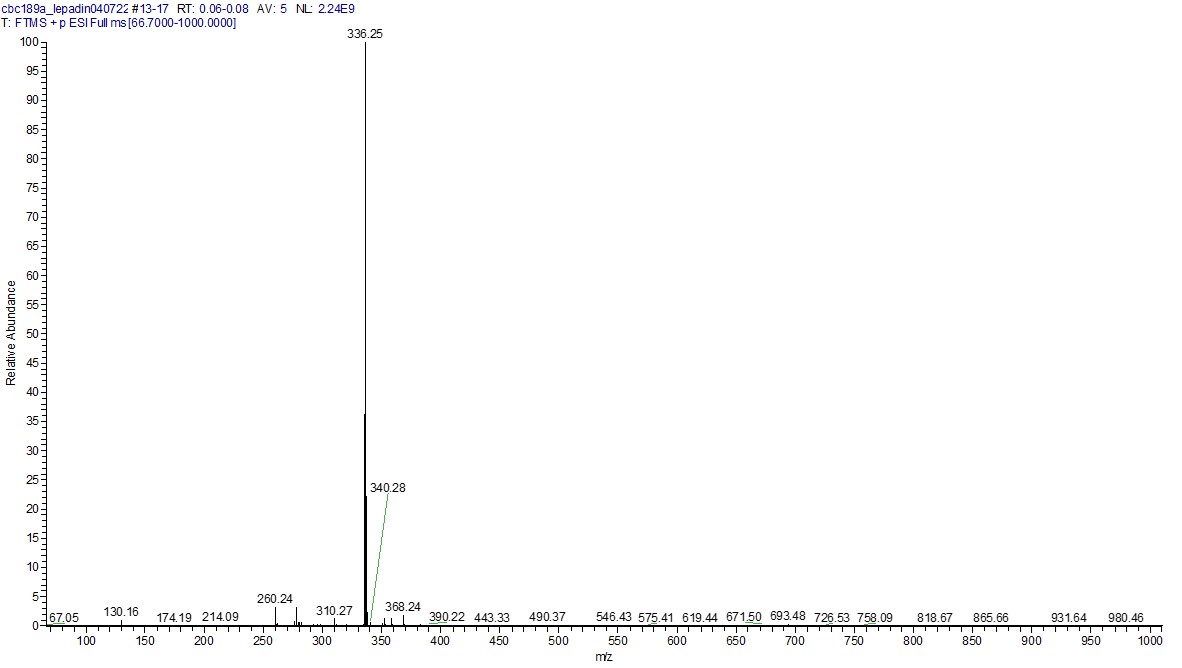
***

**Figure S2.** Exposure of CRT on A2058 cell surface to the various treatments. CRT-specific fluorescence is plotted for each gated cells (dead = blue, dying = red, live = green); (A) A2058 cells treated with doxorubicin (***2***) at EC_50_ concentration (2 µM). (B) A2058 cells treated with Cisplatin (***3***) at EC_50_ concentration (63 µM). (C) A2058 cells treated with lepadin A (***1***) at EC_50_ concentration (8 µM).

***
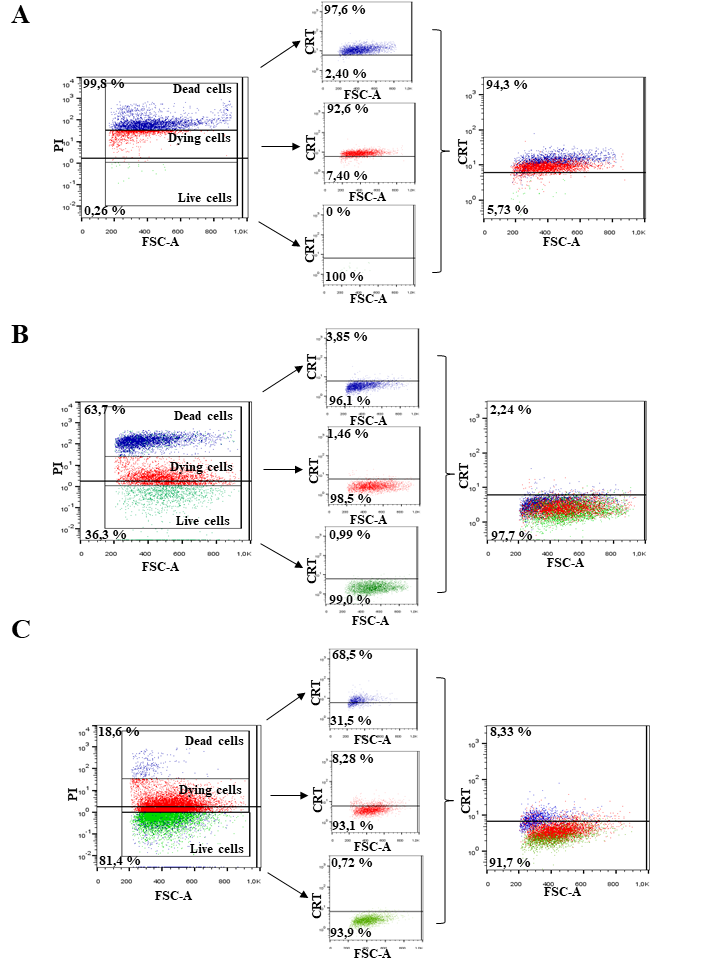
***

**Figure S3.** Gating strategy of CRT exposure. Cells were stained with 3 µg/mL of calreticulin primary antibody, with 5 µg/mL of secondary antibody (FITC) and then, with Propidium Iodide (PI). Plot A represent the physical population (SSC-A, FSC-A) corresponding to untreated A2058 cells. Plot B represents the exposure of CRT on gated A2058 of plot A, while plot C reports the PI staining. Live, dying and dead cells were gated in Plot C according to PI fluorescence intensity. CRT+ cells (D-F) were then selected from each population of plot C.


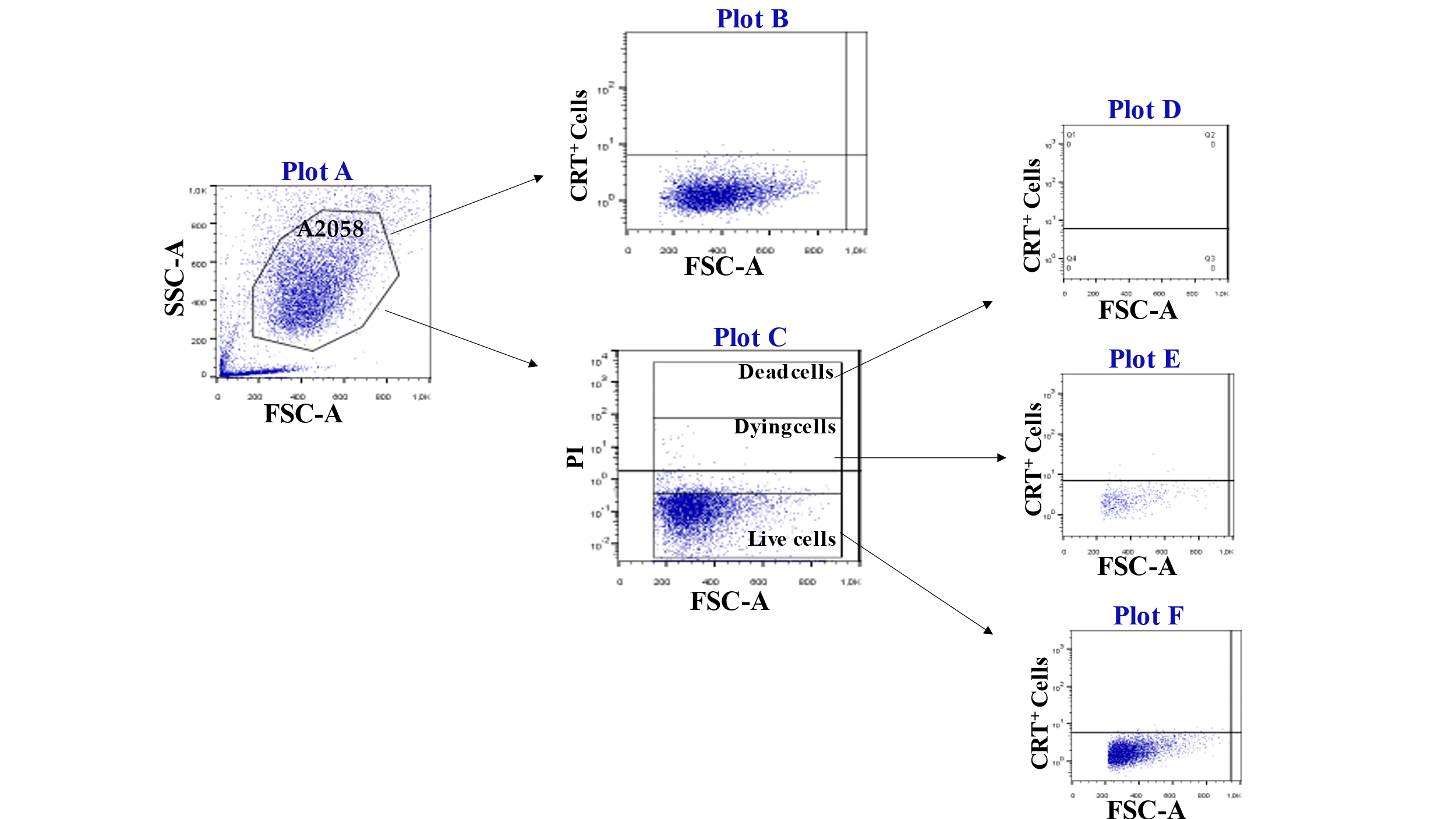


**Figure S4:** IL-1β and TNFα cytokine production (pg/mL^-1^) measured by ELISA assay. MoDCs= dendritic cells. Ctrl= MoDCs cocultured with untreated A2058 cells. (***1*)** = MoDCs cocultured with 22 µM concentration of lepadin A (***1***) pre-treated A2058 cells; (***2*)** = MoDCs cocultured with EC_50_ concentration of doxorubicin (***2***) pre-treated A2058 cells. Statistical significance (***p< 0.001, ****p<0.0001) was established by One Way Anova.


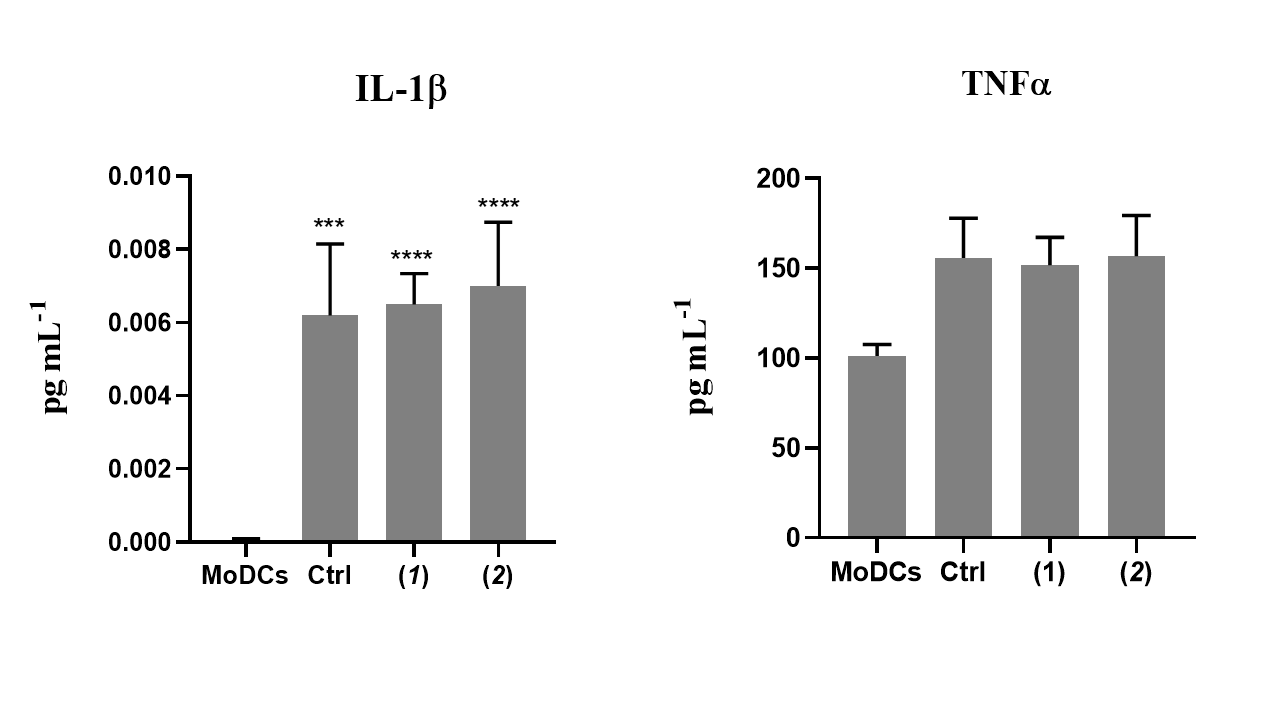


**Figure S5:** Gating strategy of MoDCs surface expression markers. Plot A represent the physical population (SSC-A, FSC-A) corresponding to MoDCs cocultured with A2058 cells. Plot B represents the exposure of HLA-DR on gated cells of plot A, MoDCs were HLA-DR + while A2058 cells were HLA-DR -. Plot C reports the Viability staining of MoDCs. CD91, CD86, CD83 (D-F) were then selected from plot C.


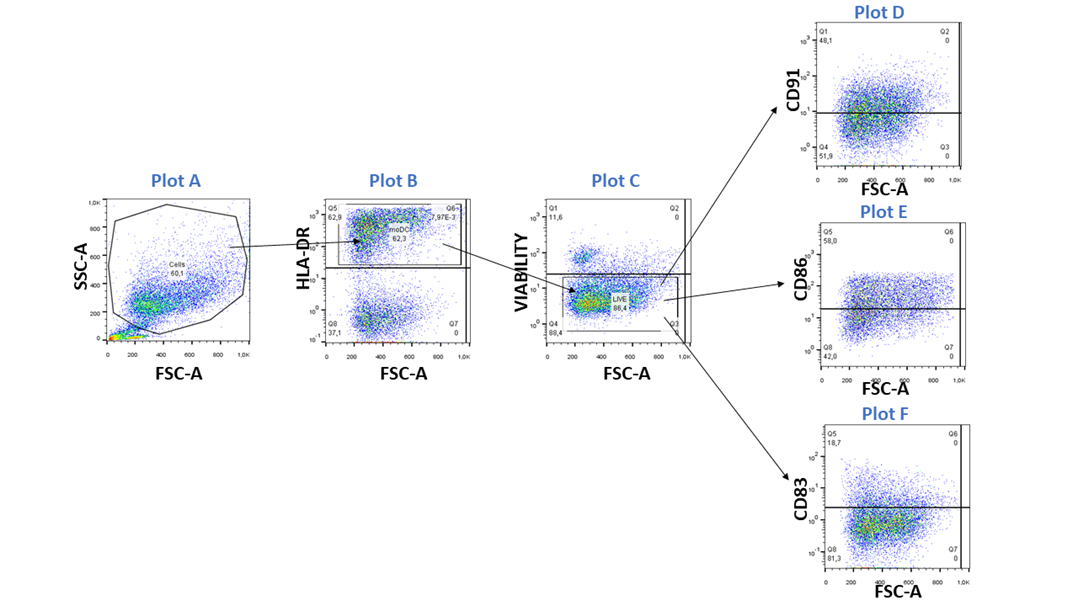

Supplement: Supplementary file 2 — Additional file 2: Figure S1. MS spectrum of lepadin A (1). The analysis was carried in positive ion mode. m/z 335, 23 (M + Na +). Figure S2. Exposure of CRT on A2058 cell surface to the various treatments. CRT-specific fluorescence is plotted for each gated cells (dead = blue, dying = red, live = green); A A2058 cells treated with doxorubicin (2) at EC50 concentration (2 µM). B A2058 cells treated with Cisplatin (3) at EC50 concentration (63 µM). C A2058 cells treated with lepadin A (1) at EC50 concentration (8 µM). Figure S3. Gating strategy of CRT exposure. Cells were stained with 3 µg/mL of calreticulin primary antibody, with 5 µg/mL of secondary antibody (FITC) and then, with Propidium Iodide (PI). Plot A represent the physical population (SSC-A, FSC-A) corresponding to untreated A2058 cells. Plot B represents the exposure of CRT on gated A2058 of plot A, while plot C reports the PI staining. Live, dying and dead cells were gated in Plot C according to PI fluorescence intensity. CRT + cells (D-F) were then selected from each population of plot C. Figure S4: IL-1β and TNFα cytokine production (pg/mL-1) measured by ELISA assay. MoDCs = dendritic cells. Ctrl = MoDCs cocultured with untreated A2058 cells. (1) = MoDCs cocultured with 22 µM concentration of lepadin A (1) pre-treated A2058 cells; (2) = MoDCs cocultured with EC50 concentration of doxorubicin (2) pre-treated A2058 cells. Statistical significance (***p < 0.001, ****p < 0.0001) was established by One Way Anova. Figure S5. Gating strategy of MoDCs surface expression markers. Plot A represent the physical population (SSC-A, FSC-A) corresponding to MoDCs cocultured with A2058 cells. Plot B represents the exposure of HLA-DR on gated cells of plot A, MoDCs were HLA-DR + while A2058 cells were HLA-DR -. Plot C reports the Viability staining of MoDCs. CD91, CD86, CD83 (D-F) were then selected from plot C. [file 13659_2023_401_MOESM2_ESM.docx]
